# Wheelchair Neuro Fuzzy Control and Tracking System Based on Voice Recognition

**DOI:** 10.3390/s20102872

**Published:** 2020-05-19

**Authors:** Mokhles M. Abdulghani, Kasim M. Al-Aubidy, Mohammed M. Ali, Qadri J. Hamarsheh

**Affiliations:** Faculty of Engineering & Technology, Philadelphia University, Amman 19392, Jordan; mukhlisalrawi@gmail.com (M.M.A.); m_selman@philadelphia.edu.jo (M.M.A.); qhamarsheh@philadelphia.edu.jo (Q.J.H.)

**Keywords:** wheelchair control, voice recognition, autonomous wheelchair, ANFIS, V-REP, mechatronics

## Abstract

Autonomous wheelchairs are important tools to enhance the mobility of people with disabilities. Advances in computer and wireless communication technologies have contributed to the provision of smart wheelchairs to suit the needs of the disabled person. This research paper presents the design and implementation of a voice controlled electric wheelchair. This design is based on voice recognition algorithms to classify the required commands to drive the wheelchair. An adaptive neuro-fuzzy controller has been used to generate the required real-time control signals for actuating motors of the wheelchair. This controller depends on real data received from obstacle avoidance sensors and a voice recognition classifier. The wheelchair is considered as a node in a wireless sensor network in order to track the position of the wheelchair and for supervisory control. The simulated and running experiments demonstrate that, by combining the concepts of soft-computing and mechatronics, the implemented wheelchair has become more sophisticated and gives people more mobility.

## 1. Introduction

The elderly, as well as millions of other people, suffer from paralysis and disability, which makes them physically unable to interact normally and adhere to the demands of life [1]. Wheelchairs are important tools to enhance the mobility of persons with disabilities. Developments in computers and communications technologies have contributed to the availability of smart wheelchairs that meet the requirements of a disabled person. In order to help the handicapped to carry out their daily work, many attempts have been made to apply modern technologies in computers and communications to build smart wheelchairs that suit their needs. These wheelchairs need to be equipped with a real-time computer control unit and a set of sensors for navigation and obstacle avoidance tasks [2,3].

A disabled person can control a wheelchair by simply moving a part of the body, using sound or brain signals. The method of generating commands for guiding the wheelchair depends mainly on the patient’s condition and degree of disability or paralysis. In our previous research [3], the brain-computer interface based on electrooculography (EOG) signals was used to control an electric wheelchair. In this paper, the voice will be used in guiding the wheelchair. 

Voice recognition has gained increasing importance in computer-controlled applications. Voice recognition techniques evaluate the voice biometrics of a person, such as the frequency, flow of voice, and accent. This technology will provide a new way of human interaction with machines. Although voice recognition is normal for people, it is not an easy task for a computer, especially when used in real-time applications. A simple design for a voice-controlled wheelchair is given in the literature [4,5,6]. The speech recognition is done by a voice recognition module connected to the main controller. The wheelchair is controlled directly by the voice commands used by an Arduino microcontroller to drive the motors. A smart phone-based, voice-controlled wheelchair is proposed by Malik et al. [5] who used an Android application to recognize a user’s voice.

Incorporating soft-computing tools, such as fuzzy logic and artificial neural network (ANN), in predicting wheelchair commands based on voice signals makes it very attractive for engineers to design and implement smart wheelchairs that suit the requirements of the disabled and elderly people [3,7]. An obstacle avoidance fuzzy controller has been used for guiding an electric wheelchair [7]. The proposed algorithm uses data from eight ultrasonic sensors distributed around the wheelchair to make navigation decisions. The power consumption was evaluated, and it was found that the field programmable gate array (FPGA) hardware implementation reduces the battery life. Wahyudi & Syazilawati [8] proposed an adaptive neuro-fuzzy inference system (ANFIS) controller for a security door access control system, to convert and classify the voice commands to control commands after feature extraction. Perceptual linear prediction coefficients with fast Fourier transform have been used as a feature of the person’s voice. Experimental results showed that the proposed system produced a good security performance. Mazo et al. [9] proposed a wheelchair control system uses dependent-user recognition voice (in generating commands) integrated with ultrasonic and infrared sensors. The wheelchair can be driven using voice commands (high-level control) and with the possibility of avoiding obstacles (low-level control). Both PID controller (for position and speed control) and fuzzy controller (for obstacle avoidance) were used in the proposed system. Xu et al. [10] present an adaptive human machine interaction method based on surface electromyography signals for the hands-free control of an intelligent wheelchair. However, the proposed detection method requires reducing noisy signals from facial movements when a user is talking and looking around.

In this research, the real-time voice recognition and intelligent control of the wheelchair are considered. The main features will be extracted from the person’s voice data and an ANFIS will be used to classify each voice command and produce the required control commands accordingly. The rest of the paper is organized as follows. The concepts of voice recognition are given in Section 2. The elements of the proposed system are discussed in Section 3. Section 4 and Section 5 present wheelchair control system design, including hardware and software design, respectively. Experimental and simulation results are discussed in Section 6. Finally, a conclusion and some suggested future work are given in Section 7.

## 2. Voice Recognition

Speech could be a useful interface to interact with machines. It has been made possible to have a system capable of real-time conversations. However, this is still facing a lot of problems, which are due to the variation in speaker due to age, gender, speed of signal, different pronunciation, surroundings noise, etc. [11,12]. In order to overcome the problems of using a joystick or any other input method needed to move muscles (especially for those suffering from a high level of disability), this paper introduces a voice-based wheelchair control system for disabled people. Voice recognition is the ability of a machine or program to receive and interpret dictation or to understand and carry out spoken commands. The first voice recognition product was launched in 1990 by Dragon. As published in the literature [9,12,13], the first voice recognition product that could recognize continuous speech was introduced by IBM in 1996. During the past twenty years, there has been exponential growth in voice-controlled applications, especially after the launch of smartphones, where more sophisticated voice recognition software products have been developed.

Voice recognition techniques are classified into two types, namely speaker dependent and speaker independent. The speaker dependent system is based on training the person who will be using the system, while the speaker independent system is trained to respond to a word regardless of who speaks. The first type demonstrates a high accuracy for word recognition, thus it is recommended for a voice-controlled wheelchair. A voice recognition unit (VRU) is required to provide communication channel between computer and human voice. This interface is mainly based on feature extraction of the desired sound wave signal. A typical voice recognition system consists of a data acquisition system, pre-emphasis of the acquired signals, feature extraction process, classification of the features, post-processing of the classifier output, and finally the control interface and device controller.

The sound signal is an electrical activity generated by the microphone. The traditional computer’s microphone was used as a voice signal reader with MATLAB software to acquire the voice signal. The computer’s microphone with the MATLAB software were used to process the detected signals and convert them into five commands, namely moving forward (Forward), moving backward (Backward), stopping (Stop), turning right (Right), and turning left (Left). These commands are used by the real-time controller to generate a sequence of control signals to adjust the speed and direction of each wheel.

## 3. The Proposed System

**Figure 1:**
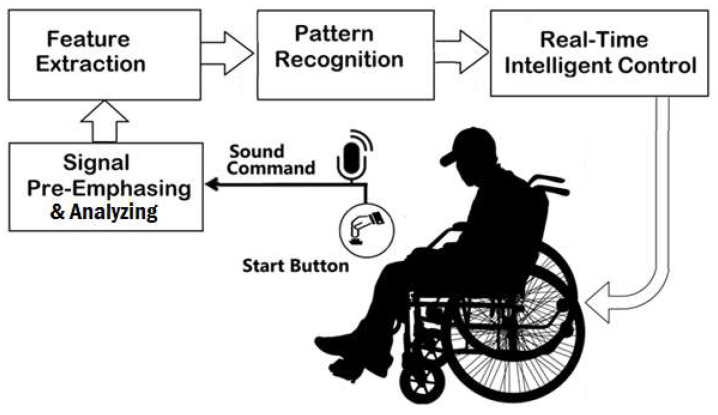
Elements of the proposed system.

The proposed system consists of four main components, namely an electric wheelchair, voice recognition unit, real-time control unit, and position tracking unit, as illustrated in Figure 1. A low-cost microphone is used as voice sensor to record the person voice. The recorded voice is then sent to the voice recognition unit, which will verify the required action, based on his/her voice. A single-chip microcontroller has been used to communicate serially with the intelligent voice recognition unit. The navigation and steering of the wheelchair has been controlled using an adaptive neuro-fuzzy inference system.

### 3.1. Electrical Wheelchair Prototype

This study contemplates an electric wheelchair prototype with two geared DC-motors. The motor actuation module has a gear ratio of 1:48 and an electronic drive module. The implemented wheelchair prototype has six ultrasonic sensors (type HC-SR04 model) to detect any obstacle and to increase the safety of motion. Two sensors were positioned at the front, two on the back, and one on each side of the wheelchair [3]. These sensors have a 2–400 cm non-contact measuring function with stable readings, and they handle good range accuracy (around 2 mm). For safety operation, the wheelchair is considered as a node in a wireless sensor network. By using this technology together with a GSM module, it becomes possible to track the position of the wheelchair and to excercise supervisory control.

### 3.2. Voice Recognition Unit (VRU)

The voice recognition unit used in this research is represented by a personal computer where MATLAB software is acquiring and classifying the voice signals received from a built-in microphone. Through MATLAB, the sound wave will be trained and classified as a command, and then these trained commands will be used via a Bluetooth module to the main microcontroller.

### 3.3. Real-Time Control Unit

The microcontroller type (MEGA-2560) has been used as the main controller. It has 54 digital input/output pins, 16 analog inputs, 8 KB SRAM, 4 KB EEPROM, and 256 KB flash memory. The microcontroller takes voice commands together with feedback signals from obstacle avoidance sensors to generate the required control signals for the driving motors.

### 3.4. Position Tracking Unit

The owner of the wheelchair can track the location and status of the wheelchair. The GSM/GPS module (type SIM808) is used to indicate the location of the wheelchair and send an SMS to the mobile phone of the owner showing the exact location on Google map application.

## 4. Hardware Design

**Figure 2:**
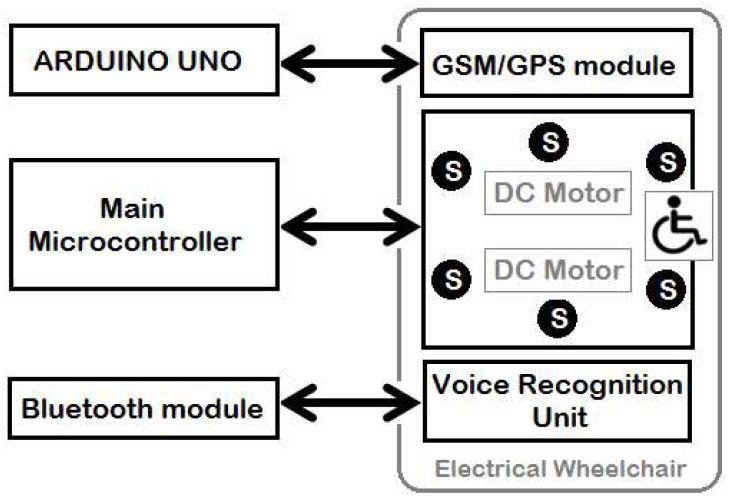
General layout of the implemented wheelchair system.

**Figure 3:**
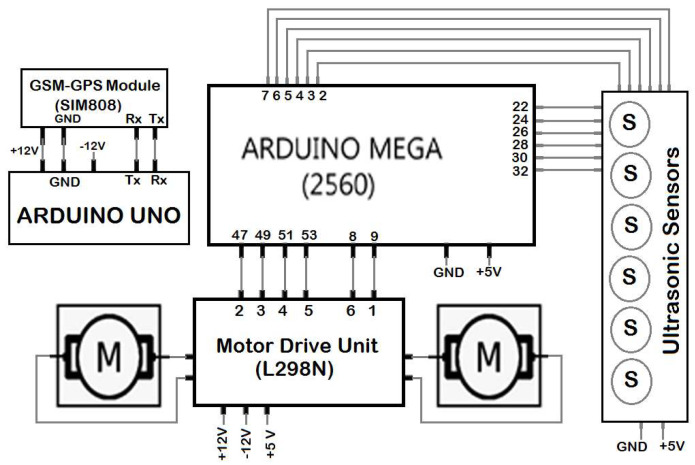
Hardware design of the wheelchair controller.

The overall layout of the hardware design of the implemented wheelchair prototype is shown in Figure 2. As shown, it has two microcontrollers, two DC motors, voice recognition unit, and six ultrasonic sensors. The voice recognition unit is connected serially to the main microcontroller via a Bluetooth module (type HC-06). An electronic drive unit (type L298N) drives each DC motor via the microcontroller. As shown in Figure 3, the main microcontroller generates the triggering signals for the six ultrasonic sensors while the output signals for these sensors are used by the real-time controller to generate the appropriate control commands (direction and duty cycle of the pulse width modulated (PWM) signal) for both right and left DC motors.

The second microcontroller type (ARDUINO UNO) is connected directly to the GSM/GPS module. It is responsible for position-tracking task and equipped with an independent power source to keep it working 24 h. The position tracking task will be managed by sending an SMS with the “track” command from the owner’s cell phone to the GSM unit. The position tracking algorithm in the UNO microcontroller responds directly by resending and texting to the owner’s cell phone with a Google Map link showing the latitude and longitude of the exact current position of the wheelchair according to the reading data of the GPS chip.

## 5. Software Design

The software module of the implemented wheelchair prototype contains three primary components, namely voice features extraction, generating control commands, and real-time controller.

### 5.1. Voice Features Extraction

**Figure 4:**
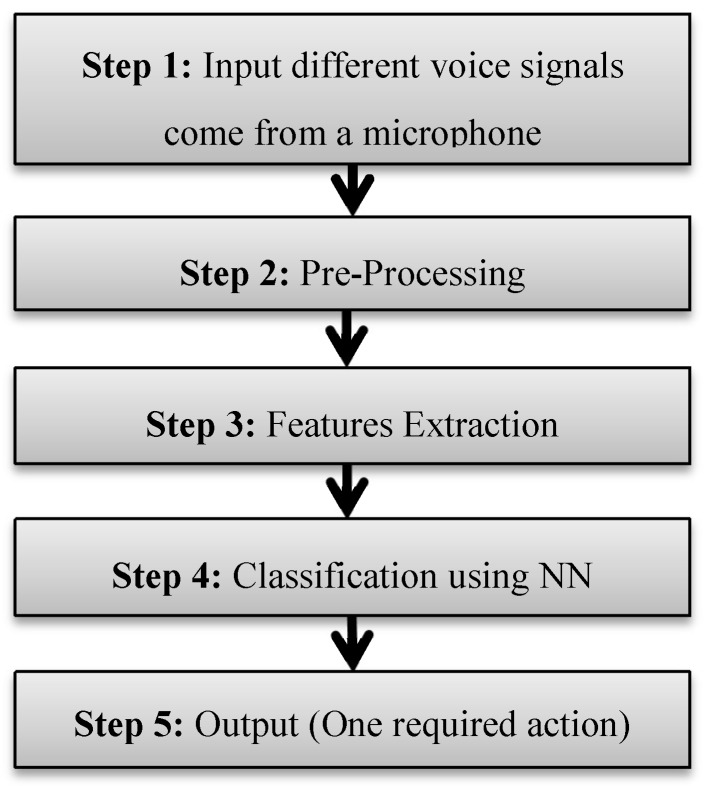
Voice recognition process.

In the feature extraction process, the raw voice signal been converted to feature vector which can be used for classification. Features are extracted from preprocessed voice and can be used to represent the voice signal. In general, speech recognition is mainly done in two stages, namely training and testing. However, before this, some basic procedures are necessary applied to speech signals. Figure 4 outlines the basic process of speech recognition. It shows that an input of different voice signals come from a microphone before it is preprocessed using suitable techniques like filtering. The regarding useful features are extracted to distinguish between different signals [13]. In this research, the classification process is achieved using neuro-fuzzy controller. A neural network (step 4) is trained based on the selected features extracted (step 3) from the input speech signals (step 1).

*Pre-emphasis (Step 2):* In this step an equal loudness curve is constructed. Each channel (with 80 samples per frame) has been filtered independently using a finite impulse response filter. This filter emphasizes high frequencies and attenuates lowers. The overlap analysis block is used to convert scalar samples to a frame output at a lower rate. Then, the voice data are framed and windowed using the available window function such as hamming window.

**Figure 5:**
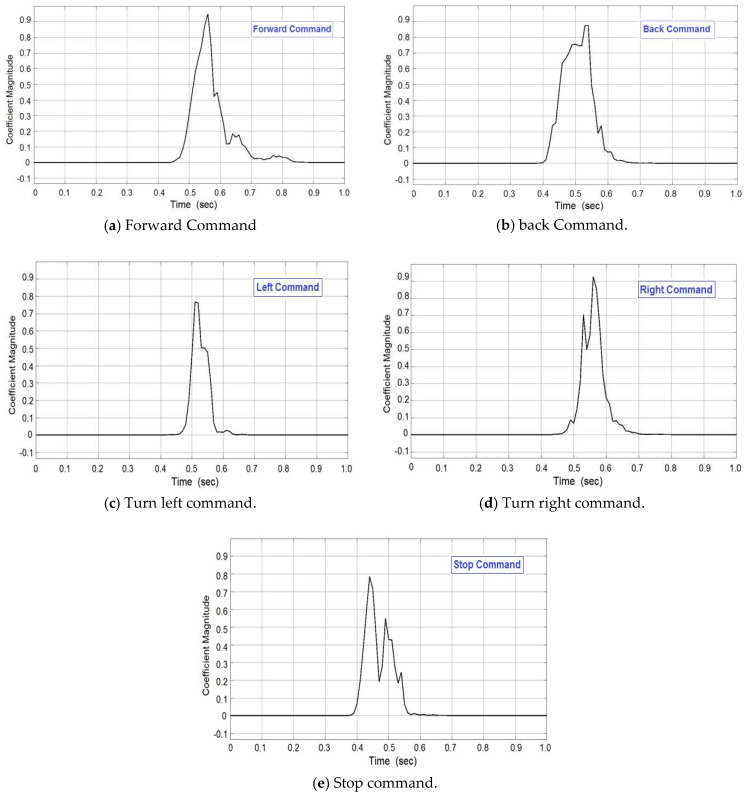
Autocorrelation coefficient of the voice commands.

*Autocorrelation signal:* It is a mathematical tool for finding repeating patterns by calculation of the all-pole coefficients. Autocorrelation can be used to calculate the all pole coefficients using the well-known “Levinson–Durbin” algorithm [8]. Using the MATLAB Simulink, autocorrelation has been done for the selected five voice commands, namely Forward, Back, Left, Right, and Stop, as given in Figure 5. Correlation signal analysis has been achieved between signals (frames) of the given class (Forward, Backward, Right, Left, and Stop). The results of correlation analysis showed the possibility of using these signals to implement feature extraction (step 3).

**Figure 6:**
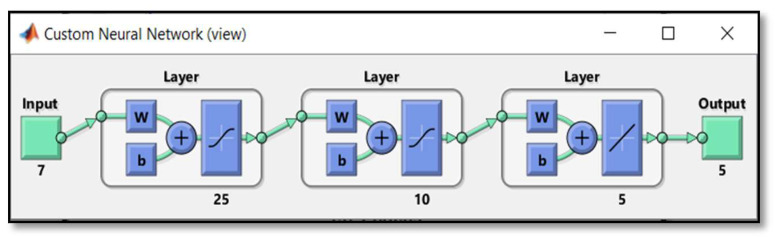
The implemented NNs Topology.

*Neural networks controller design:* In this step, different voice signals (80 frames for each action direction Stop, Forward, Back, Right, and Left) are taken from the recorded input speech signals. Two data sets, one for training and the other for validation and testing are chosen based on seven statistics features (Mean, Median, Minimum, Mode, Peak-to-Peak, RMS, and Standard Deviation). The dimension of the training and testing input matrices is of (7 × 400) each. While the target data is a matrix of (5 × 400) dimension. The classification has been made using neural network tool on MATLAB version R20116a workspace. The implemented neural network topology was of (7-25-10-5). It has a 7-node linear input layer, two sigmoidal nonlinear hidden layers of 25 and 10 units respectively, and a 5-node linear output layer as shown in Figure 6.

**Figure 7:**
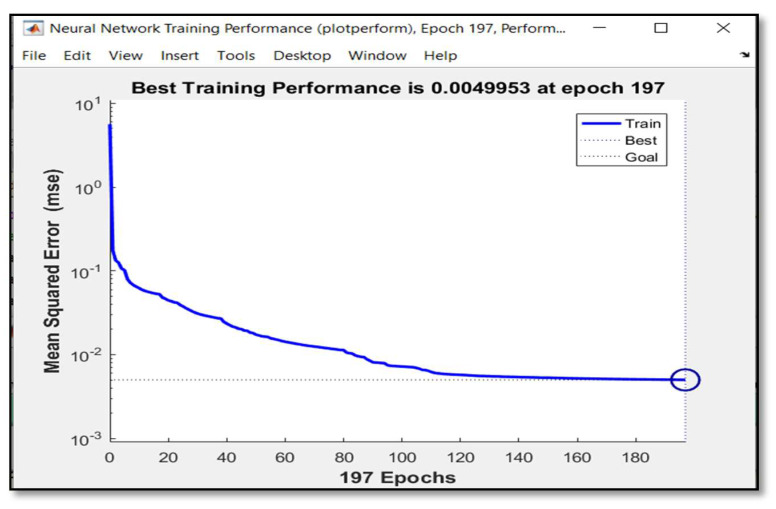
Performance of neural networks.

An error-back propagation learning algorithm has been applied based on a Levenberg–Marquardt algorithm with learning rate of 0.05 and stopping criterion of mean error square less than or equal to 0.005. As illustrated in Figure 7, after 197 iterations, the neural network has learnt effectively.

The tested data is used to confirm learning with an output of the required action signal is perfectly achieved, as shown in Table 1 and Table 2 as a sample. The seven selected features of each voice commands given in Table 1 are used for training the neural network to recognize each command. Meanwhile, Table 2 shows the five required outputs for each voice commands which will be implemented by the main microcontroller.

### 5.2. Generating Control Commands

As given in Figure 4, step 5 is dedicated to convert the trained and classified sound commands to control commands using the ANFIS. Five control commands are considered, namely moving forward (Forward), moving backward (Back), stopping (Stop), turning right (Right), and turning left (Left).

### 5.3. Real-Time Control

The implementation of fuzzy logic as a decision tool and artificial neural network as a modeling methodology will help designers to investigate controllers without the need for accurate mathematical model of the plant to be controlled. Therefore, these soft-computing tools open the way for new researches for the real-time control of an intelligent wheelchair. For safe mobility and smooth steering of the wheelchair, the MATLAB neuro-fuzzy design application has been used to construct an ANFIS to calculate the accurate duty cycle of the PWM signal sent to each DC motor. The direction and the speed of rotation for each wheel will be controlled by the duty cycle value of the PWM signal. The duty cycle value (100) has been selected to set the maximum speed of the wheelchair.

**Figure 8:**
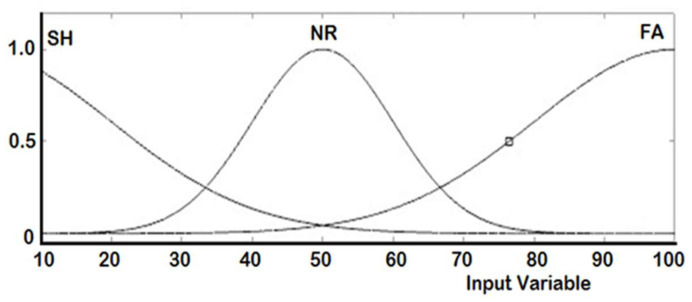
Membership functions before training.

**Figure 9:**
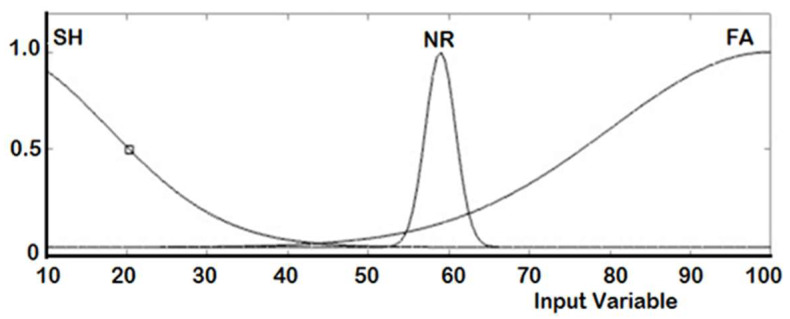
Membership functions after training for the right DC-motor output.

**Figure 10:**
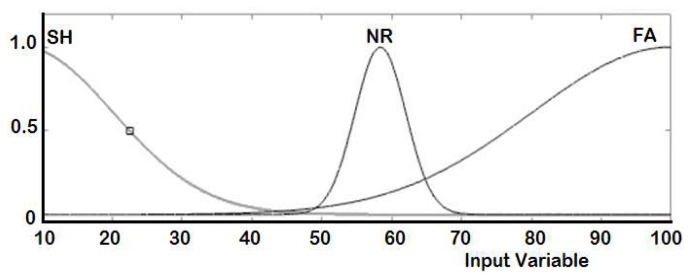
Membership functions after training for the left DC-motor output.

The real-time controller reads the output of the six Ultrasonic sensors (S_1_ to S_6_) in centimeter and accordingly generates the duty cycle for each PWM signal to drive the right and the left DC motors. Two ANFIS controllers are designed, one for each DC motor. Table 3 shows the training dataset used in the learning process implemented by the ANFIS. The measured distance generated from each ultrasonic sensor is represented by three fuzzy sets with Gaussian membership functions. These fuzzy sets are short (SH), normal (NR), and far (FA), as illustrated in Figure 8. The ANFIS is used to tune the membership functions of the fuzzy sets for both right and left motors are given in Figure 9 and Figure 10, respectively.

**Figure 11:**
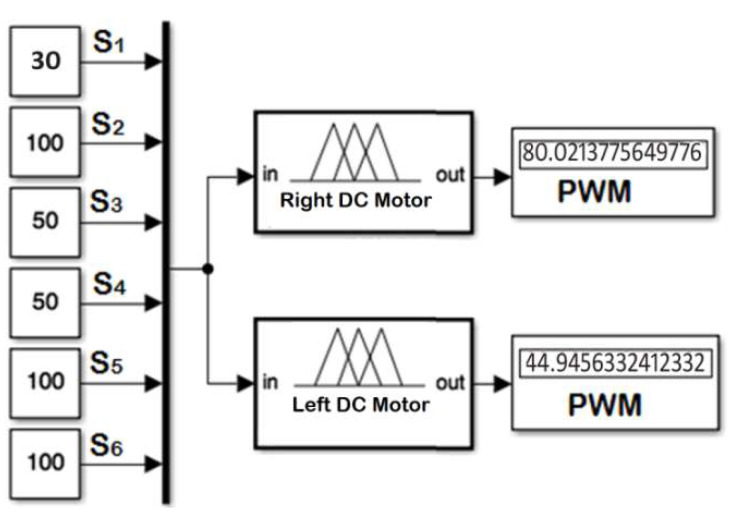
MATLAB Simulink output test for the ANFIS controller.

The resulting multi-input multi-output (MIMO) ANFIS algorithm given in Figure 11 has been tested on the simulation model and the real prototype. The performance of the resulting MIMO ANFIS algorithm was perfect and all the cases have been covered—even the in-between cases have been covered extremely perfect. Table 4 shows the dataset and generated values of the duty cycles of the pulse width modulated (PWM) signals of both right and left DC motors using the neuro fuzzy controller. It is clear that the error between generated and desired root mean square error (RMSE) values of the PWM signals are 0.082 for right wheel and 0.339 for left wheel.

## 6. Results and Discussion

**Figure 12:**
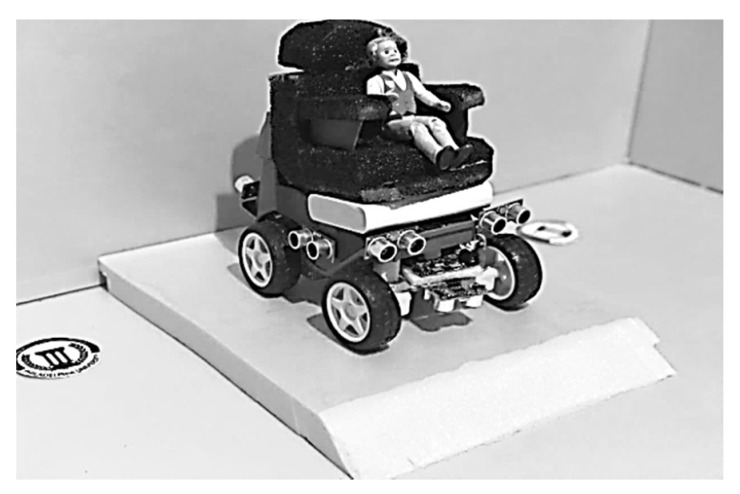
The implemented wheelchair prototype.

The principal part of the software implemented in this research work is the extraction of voice features. The implemented software enables the voice signals to be read and processed from a built-in microphone into command. It sends the command signal over a Bluetooth connectivity module to the microcontroller. The real-time controller produces the control signals needed for both the right and left motors. For safe operation, the maximum speed of the implemented wheelchair system, as shown in Figure 12, is 125 rpm, when the PWM signal duty cycle is only 40% of the full value.

**Figure 13:**
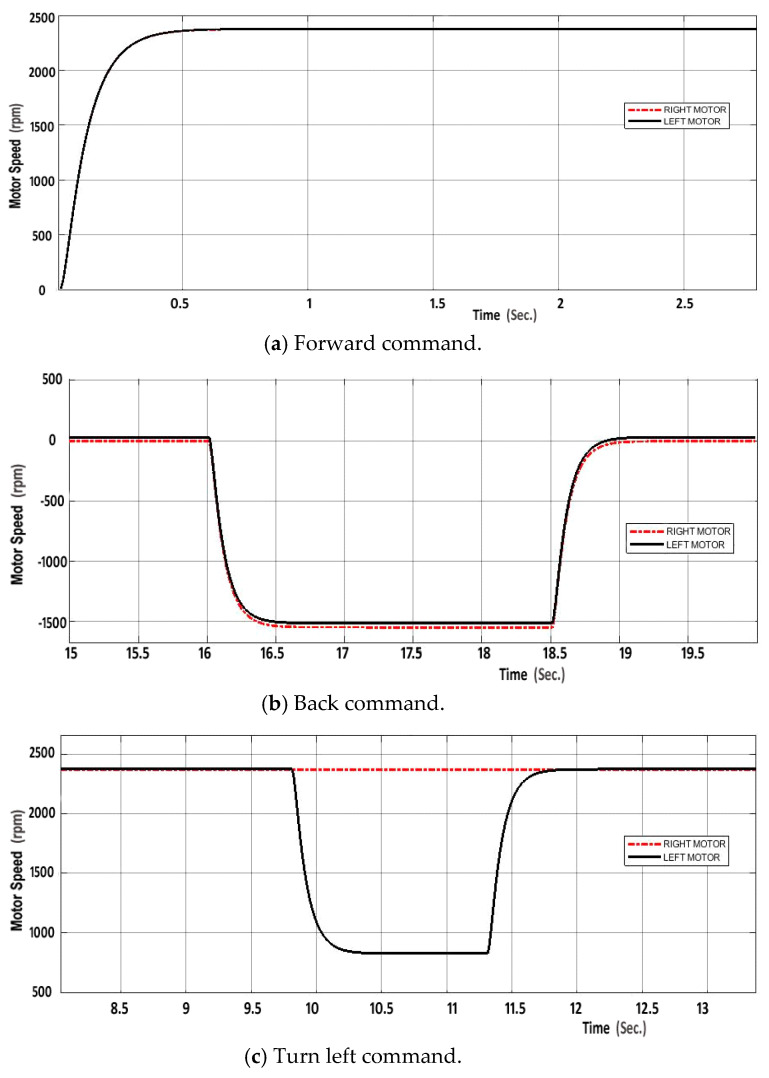

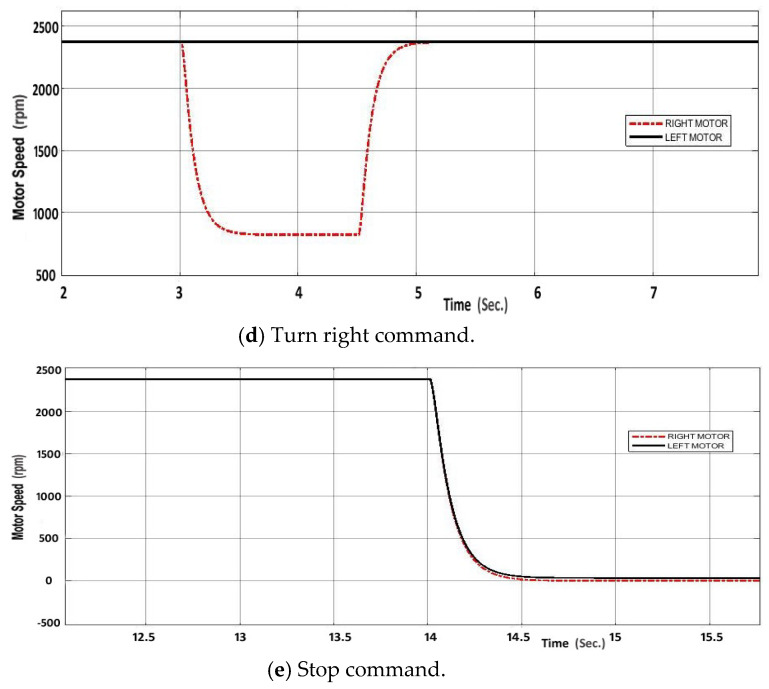
Speed responses of two DC motors for different commands.

A real-time simulator was developed that integrates knowledge about the wheelchair and its working environment to illustrate wheelchair actions and how it will act according to the voice commands. The speed responses for both left and right motors to the five commands provided by the voice recognition module are demonstrated in Figure 13.

**Figure 14:**
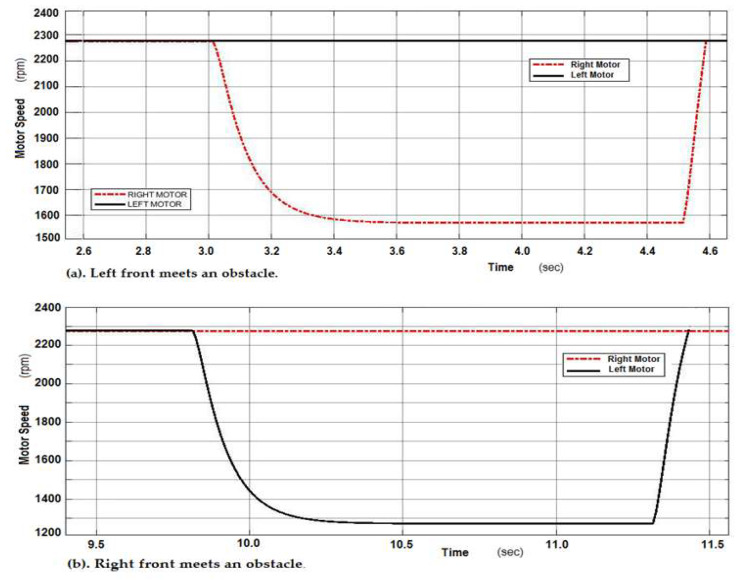
Speed responses of two DC motors during obstacle avoidance.

The ANFIS controller’s actions has been evaluated and tested when an obstacle appears in the wheelchair’s working area. Figure 14 illustrates the speed responses of both motors when the wheelchair on the left front meets an obstacle. It is obvious that the speed of the right motor is reduced to allow the wheelchair to turn right to avoid obstacles. If the wheelchair meets an obstacle on the right front side, the speed of the left motor is reduced to enable the wheelchair to turn left.

**Figure 15:**
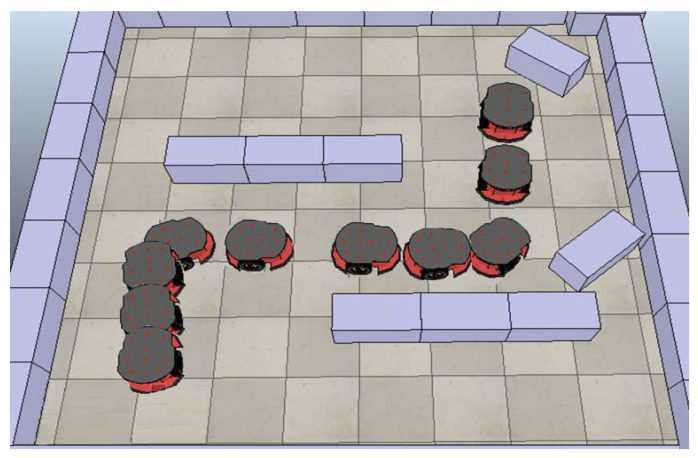
V-REP simulation during obstacle avoidance.

The direct interface between MATLAB Simulink, and the V-REP 3D simulator software is an approach to simulate the behavior of the implemented wheelchair system. Figure 15 illustrates the behavior of the 3D simulation model during the implementation of the resulting MIMO ANFIS algorithm. It is clear that the wheelchair model is able to avoid obstacles on the left and right front sides. The MIMO ANFIS controller is able to make the required decision, even with obstacle distance excluded from the training data given in Table 4.

A supervisory control mode can be used via the GSM technique, whereby the wheelchair receives control commands from the owner by sending SMS to the wheelchair, such as to stop the wheelchair or move it in any direction. The owner can send SMS with the command “check” and the wheelchair system will reply immediately with SMS showing the status of the wheelchair (location & battery level). Moreover, once the stop command been activated to stop the wheelchair, a timer will start counting time, if the timer reaches three minutes and no forward action been executed, an emergency SMS will be sent to the owner telling him that the wheelchair is stopped for more than three minutes and the patient or the user might be in a trouble or might be in a sleeping situation. More safety consideration has been included using the GSM/GPS technique. The second microcontroller (ARDUINO UNO) was programmed to respond to the SMS commands received from the wheelchair’s owner. In this case, the wheelchair’s owner sends an SMS message with the word “track” and then immediately receives an SMS response from the second microcontroller. Using such a technique will update the location and the battery level situation for the owner by sending an SMS each 15 or 20 min, or any time could be indicated depending on the patient’s situation, to inform him the location of the wheelchair located and what is the battery level.

## 7. Conclusions

An ANFIS based voice-controlled wheelchair was designed and implemented to support individuals with physical disabilities. By using voice instructions, the patient can control the electrical wheelchair. The functioning and overall performance of the implemented wheelchair prototype system was tested using various test commands and perturbations. The results obtained from the simulator and prototype model demonstrate that the use of the ANFIS based controller together with online sensor signals can maximize wheelchair performance and improve the quality of life of physically challenged people. The implemented prototype has many benefits, including simplicity, inexpensive, position tracking, and safety. It has a set of sensors to detect static and dynamic obstacles as well any slippery roads.

A feed-forward multilayer neural network with (7-25-10-5) topology of input, hidden and output layers was implemented for classification to recognize the voice of individual speakers with suitable datasets for training and testing.

## Figures and Tables

**Table 1:** A sample of test input pattern.

Testing Input Pattern					
Features	Stop	Forward	Back	Right	Left
Mean	−0.008	−0.0075	0.0056	−0.0006	0.0048
Median	−0.002	0.00015	−0.00006	0.00018	0
Minimum	−0.21	−0.3176	−0.05	−0.1021	−0.0313
Mode	0	−0.0002	−0.00006	0	0
Peak to Peak	0.4356	0.4716	0.2078	0.2809	0.286
RMS	0.0468	0.0615	0.0284	0.024	0.03
STD	0.0463	0.0614	0.028	0.024	0.0302

**Table 2:** Target and actual NNs output for given input pattern.

Output Pattern		
Action	Target	Actual NNs Output
Stop	1	1.012
	0	−0.002
	0	0.018
	0	−0.025
	0	−0.002
Forward	0	−0.0008
	1	1.0014
	0	−0.0005
	0	−0.00002
	0	−0.00007
Back	0	0.0058
	0	−0.0002
	1	1.0262
	0	−0.004
	0	−0.0283
Right	0	−0.0117
	0	0.0001
	0	0.0308
	1	1.0313
	0	−0.0507
Left	0	−0.01
	0	0.0002
	0	0.018
	0	0.0014
	1	0.99

**Table 3:** Dataset used for training the real-time controller.

Sensors Outputs (Cm)						PWM Duty Cycle	
S_1_	S_2_	S_3_	S_4_	S_5_	S_6_	Right Motor	Left Motor
100	40	50	50	100	100	70	100
30	100	50	50	100	100	80	45
100	100	20	50	100	100	100	90
100	100	40	10	100	100	65	80
40	50	15	100	100	100	80	55
40	45	100	20	100	100	55	75
40	40	25	25	100	100	65	20
100	100	40	40	35	100	100	80
100	100	40	40	100	35	80	100

**Table 4:** Resulting control signals for the same trained dataset.

Sensors Outputs						PWM Duty Cycle	
S_1_	S_2_	S_3_	S_4_	S_5_	S_6_	Right Motor	Left Motor
100	40	50	50	100	100	69.96	99.89
30	100	50	50	100	100	80.02	44.94
100	100	20	50	100	100	99.82	90.01
100	100	40	10	100	100	64.99	80.15
40	50	15	100	100	100	79.98	55.1
40	45	100	20	100	100	55.12	74.88
40	40	25	25	100	100	64.99	20.05
100	100	40	40	35	100	100.13	80.05
100	100	40	40	100	35	79.87	99.9
